# Development of silicon-on-insulator direct electron detector with analog memories in pixels for sub-microsecond imaging

**DOI:** 10.1093/jmicro/dfae029

**Published:** 2024-06-01

**Authors:** Takafumi Ishida, Kosei Sugie, Toshinobu Miyoshi, Yuichi Ishida, Koh Saitoh, Yasuo Arai, Makoto Kuwahara

**Affiliations:** Institute of Materials and Systems for Sustainability, Nagoya University, Furo-cho, Chikusa, Nagoya 464-8601, Japan; Graduate School of Engineering, Nagoya University, Furo-cho, Chikusa, Nagoya 464-8601, Japan; Graduate School of Engineering, Nagoya University, Furo-cho, Chikusa, Nagoya 464-8601, Japan; Institute of Particle and Nuclear Studies, High Energy Accelerator Research Organization (KEK), 1-1 Oho, Tsukuba 305-0801, Japan; School of Engineering, Nagoya University, Furo-cho, Chikusa, Nagoya 464-8601, Japan; Institute of Materials and Systems for Sustainability, Nagoya University, Furo-cho, Chikusa, Nagoya 464-8601, Japan; Graduate School of Engineering, Nagoya University, Furo-cho, Chikusa, Nagoya 464-8601, Japan; Accelerator Laboratory, High Energy Accelerator Research Organization (KEK), 1-1 Oho, Tsukuba 305-0801, Japan; Institute of Materials and Systems for Sustainability, Nagoya University, Furo-cho, Chikusa, Nagoya 464-8601, Japan; Graduate School of Engineering, Nagoya University, Furo-cho, Chikusa, Nagoya 464-8601, Japan

**Keywords:** SOI pixel detector, back-side illumination, high-speed imaging, pulsed electrons

## Abstract

We have developed a high-speed recordable direct electron detector based on silicon-on-insulator technology. The detector has 16 analog memories in each pixel to record 16 images with sub-microsecond temporal resolution. A dedicated data acquisition system has also been developed to display and record the results on a personal computer. The performance of the direct electron detector as an image sensor is evaluated under electron irradiation with an energy of 30 keV in a low-voltage transmission electron microscope equipped with a photocathode electron gun. We demonstrate that the detector can record images at an exposure time of 100 ns and an interval of 900 ns.

## Introduction

The development of direct electron detectors (DEDs) has played a role in improving the quality of images acquired using transmission electron microscopes [1,2]. In particular, DEDs are critical for observing biological and organic specimens via transmission electron microscopy (TEM) [3,4] because such specimens require low-dose, high-resolution and high-speed imaging to reveal their fine structures. In addition, DEDs, which have single electron counting capability, are useful for ultra-fast imaging by time-resolved transmission electron microscopy (TR-TEM) [5] because the improvement in temporal resolution is usually accompanied by a decrease in the number of detected electrons. The single electron counting capability of DEDs will also lead the signal-to-noise ratio of images to the theoretical limit in TR-TEM. The usefulness of DEDs stems from their high detection efficiency, high modulation transfer function (MTF) and high read-out speed compared with those of traditional detectors with scintillators, such as charge-coupled device cameras [6]. Their high detection efficiency originates from the high number of generated charges at the sensor layer by primary electrons with high energy. Their high MTF was realized by thinning the sensor thickness and post-processing based on single electron counting. On the other hand, their read-out speed depends on the read-out method, and most DEDs are based on complementary metal oxide semiconductor (CMOS) technology.

The read-out speed of DEDs depends on the number of pixels and restricts their frame rates. The number of parallel read-outs from the detectors is limited by the performance of data acquisition (DAQ) systems, which consist of read-out circuits and a personal computer (PC). The frame rate of commercial DEDs decreases with increasing pixel number [7–9]. For example, the minimum repetition time, which corresponds to the frame period, is approximately 0.5 ms at 1 Mpixels (1024 × 1024 pixels) [8]. Current DEDs cannot be used to observe nonreversible phenomena that occur on a sub-microsecond timescale.

We can overcome the frame rate limitation of the DAQ system by using a special chip design. In optical cameras, a frame rate greater than 100 Mfps has been achieved by Suzuki et al. [10], who incorporated a memory array into the sensor chip. This principle can be briefly explained as follows: The signal obtained in each pixel is quickly transferred to the memory array on the chip, and the signal is then slowly read out from the chip by a DAQ system. The aforementioned frame rate limitation is avoided by preserving the signal in the chip, and the recording length of the number of frames is restricted by the density of the memory array in the chip. Unfortunately, adapting a high-frame-rate optical camera to a DED is difficult in terms of radiation hardness against high-energy electrons [11].

We have used silicon-on-insulator (SOI) pixel detectors [12] as DEDs [13] because they are expected to exhibit radiation hardness in a transmission electron microscope. The SOI pixel detectors are monolithic radiation beam imaging sensors that can detect electrons, photons, neutrons, etc. [13–17]. Recent SOI pixel detectors have a radiation hardness of 100 kGy [18,19], which satisfies a criterion of Faruqi et al. [11]. Compared with the conventional CMOS technology, the SOI–CMOS technology has the advantages of high speed and low noise because of low parasitic capacitance [20]. We therefore expect the SOI pixel detector to be the next-generation DED. In a previous study [17], an SOI pixel detector with three analog memories was developed as a vertex detector for the international linear collider project. The number of memories, however, is insufficient for recording high-speed phenomena in electron microscopy. In this study, we report the details of a prototype SOI pixel detector with 16 analog memories in each pixel; this detector is based on the above-mentioned concept. We also report its dedicated DAQ system for sub-microsecond imaging. We further demonstrate that the prototype SOI pixel detector can record images with a sub-microsecond frame period.

## SOI device and experimental setup

### SOI pixel detector with analog memories

**Fig. 1.**
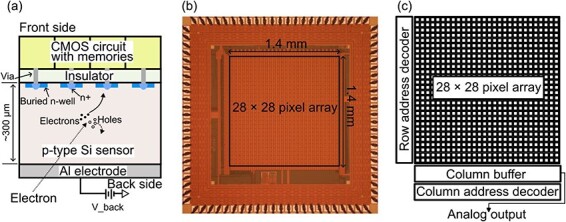
An SOI pixel detector with analog memories. (a) A schematic of the cross-section of the SOI pixel detector. Figure (a) is not the exact size of the cross-section. (b) An optical micrograph of the SOI pixel detector chip and (c) a schematic of the chip layout.

We have developed a novel SOI direct electron detector, named the silicon-on-insulator for time-resolved imaging in electron microscopy (STRIEM), which is capable of sub-microsecond imaging. Figure 1 shows a cross-sectional schematic of the STRIEM, an optical micrograph image of the STRIEM ver. 1 (hereafter called STRIEM1) chip and a schematic of the chip layout. The structure of the STRIEM (Fig. 1(a)), in which the CMOS circuit and the sensor layers are bonded through the insulator, is the same as that of conventional SOI pixel detectors. The chip shown in Fig. 1(b), which has one polysilicon and five metal layers, was fabricated in a 0.2-μm SOI–CMOS process by Lapis Semiconductor Co., Ltd. The wafer for the sensor was a p-type silicon wafer with a high resistivity of ∼5 kΩ· cm. The sensor layer was thinned to 300 μm, followed by deposition of aluminum to a thickness of 200 nm. The aluminum layer functions as an electrode for the application of a bias voltage used to fully deplete the sensor layers. Here, the full depletion voltage of the sensor layer was estimated at approximately −180 V from the resistivity and the thickness. The chip had 28 × 28 pixels within a 1.4 × 1.4 mm^2^ area (pixel size 50 × 50 μm^2^) with peripheral circuits. The main peripheral circuits are address decoders to read out a pixel value and a column buffer to connect the pixels to an output terminal (Fig. 1(b)).

**Fig. 2.**
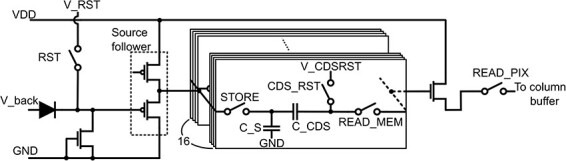
A schematic of the pixel circuit of the SOI pixel detector with analog memories (STRIEM1). The reset voltage and the CDS reset voltage are displayed as V_RST and V_CDSRST, respectively.

Figure 2 shows a pixel circuit of the SOI DED with analog memories (STRIEM1). The switch and input voltage names are displayed near these elements. Each pixel has 16 capacitors (C_S) consisting of metal-insulator-metal (MIM) capacitors in the metal layers to recode the detected voltage with a correlated double sampling (CDS) circuit (the switch CDS_RST and the capacitor C_CDS) that can cancel the reset noise. Electrons irradiating the detector induce the formation of electron-hole pairs at the sensor layer, which is a fully depleted photodiode. The generated electrons are collected in the cathode of the photodiode. The voltage of the node after the cathode, which is changed by the collected electrons, is stored at a capacitor (C_S) through a source follower and the STORE switch. The voltage of the capacitor is fed to the column buffer using the READ_MEM and the READ_PIX switches. An exposure sequence is described in the next section.

### Data acquisition system and an exposure sequence

**Fig. 3.**
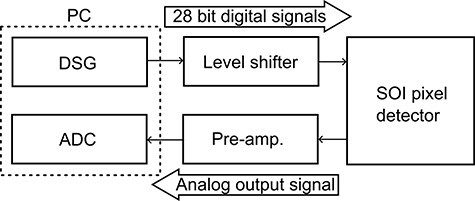
A schematic of the DAQ for STERIM1.

We have also developed a dedicated DAQ system with a data readout circuit and software to control switches for STRIEM1. Figure 3 shows a schematic of the DAQ system. Digital signal generator (DSG; DIO-32DM3-PE, CONTEC) and analog-to-digital converter (ADC; DIG-100M1002-PCI, CONTEC) boards were installed in a PC. These boards were controlled by original graphical user interface software developed using LabVIEW. Twenty-eight-bit digital signals generated by the DSG to control the CMOS switches of STRIEM1 were converted from low-voltage transistor transistor logic (3.3 V) signals to 1.8 V signals by a level shifter. These signals were then sent to STRIEM1. Analog signals in pixels were read out through a pre-amp in sequence and recorded by the ADC. Here, the pre-amp was added to support the voltage drop in the chip, which was caused by the mismatch of impedance between the column buffer and the analog output buffer. This issue has been fixed in STRIEM ver. 2. While the minimum clock period of the DSG was 20 ns, the minimum exposure and rest time for the DAQ system during stable operation, which depends on the PC and the software, was 100 ns.

**Fig. 4.**
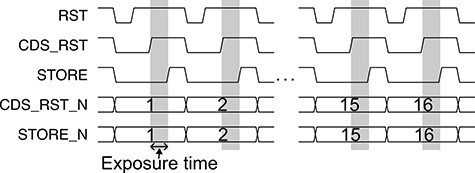
A timing diagram of an exposure sequence using 16 analog memories. Exposure times are highlighted in gray areas. The timing diagram is displayed by negative logic.

Figure 4 shows a timing diagram of an exposure sequence using 16 analog memories. The digital signal names in Fig. 4 correspond to the switch names in Fig. 2. To record images, we first initialized a memory, which corresponded to an analog capacitor (C_S), by turning on the reset switch (RST) and the store switch (STORE). The CDS reset switch (CDS_RST) was also turned on at the same time. The CDS_RST was then turned off after the RST because the CDS circuit reduced the reset noise from the signal. Finally, the STORE was turned off and a frame was obtained. The exposure time was the difference between the CDS_RST and STORE signals. To record a series of 16 frames, signals of CDS_RST_N and STORE_N, which appoint a recording memory, were changed to the next frame memory synchronized with the RST signal.

### Experimental setup for performance evaluation

We installed STRIEM1 in the camera chamber of a time-resolved transmission electron microscope [21] to evaluate the performance of STRIEM1 as a high-speed imaging sensor. The time-resolved transmission electron microscope was operated at an accelerating voltage of 30 kV and was equipped with a semiconductor photocathode electron gun, which can emit electrons by laser irradiation. The optical system for laser irradiation of the photocathode consisted of a continuous-wave (CW) laser and an acoustic optical modulator (AOM) with several mirrors. The optical system was the same as that used in previous studies [22]. The AOM can generate laser beams from CW to pulse beams up to ∼100 ns of the pulse width with an input electrical signal controlled by the DSG.

The performance of STRIEM1 with the DAQ system was evaluated using a time-resolved transmission electron microscope. We evaluated the imaging performance by obtaining an image of a binary slit using electron beams and observing the sharpness of the obtained image. The binary slit, which consisted of a Mo thin disk with a thickness of 10 μm, was fabricated with a focused ion beam (FIB) instrument (FB-2100, Hitachi High-Tech). The pattern of the slit was the letters ‘SOI’, and the letter ‘O’ had two bridges to support the central obstruction. The timing of the electron beam irradiation of STRIEM1 was matched with the start of the exposure. We also confirmed the feasibility of high-speed recording of images at the sub-microsecond timescale with STRIEM1. To test the high-speed recording, we generated pulsed electron beams and captured the pulsed electron beams in 16 frames. The pulsed electron beams were converged at the detector plane using projector lenses to visualize their time variation. We subtracted the background signal from the obtained images using each dark image. The back-bias voltage (V_back) of STRIEM1 was set to −250 V, which was sufficiently more negative than the full depletion voltage, in each experiment. Notably, the electron beams were detected at the sensor layer of STRIEM1, which corresponds to back-side illumination, directly. Therefore, the primary electrons at 30 keV, which have a penetration depth of under 10 μm for silicon calculated by the Kanaya-Okayama formula [23], cannot reach the CMOS circuit layer.

## Results and discussion

### TEM imaging using 30 keV electrons

**Fig. 5.**
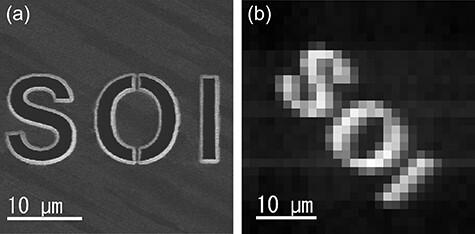
Microscope images of the letters ‘SOI’. (a) A scanning ion microscope image obtained with the FIB instrument. (b) A TEM image obtained with STRIEM1. The size of the scale bars corresponds to the size of the specimen plane.

We acquired a TEM image with STRIEM1 to confirm that it functions as an image sensor. The observation sample was a binary slit (Fig. 5(a)). Figure 5(b) shows a TEM image of the slit, as obtained with STRIEM1. The exposure time of the TEM image was 300 μs, and the image integrated 100 frames to reduce the shot noise. The TEM image clearly shows the letters ‘SOI’. Here, the letters in Fig. 5(b) are rotated 45° clockwise because of the magnetic lenses for the electrons. The result indicates that STRIEM1 can function as an image sensor with direct detection.

To estimate the sharpness of the image obtained with STRIEM1, we roughly measured the line spread function (LSF) from Fig. 5(b). When an electron beam enters a single pixel with surrounding pixels, blurring of the image occurs because of charge-sharing in the sensor layer. The blur corresponds to the LSF of the detector. Considering a simple model for the blur, we can use a Gaussian function for least-squares fitting of the blur. Four line profiles were extracted from the letter ‘O’ near the bridges because the width of the slits nearly corresponds to the pixel size at the current magnification. The full-width at half-maximum (FWHM) of the LSF was estimated to be 1.9 ± 0.1 pixels from the fitting results of the line profiles. This width is in good agreement with that for the integration-type SOI pixel detector INTPIX4 [13]. The LSF indicates that two-point objects can be observed when the image has an interval of one pixel on the detector plane. This result also implies that STRIEM1 has adequate resolving power for 30 keV electrons. We note that precise measurements of the LSF and the MTF should be performed using the slanted edge method.

### Exploring the minimum repetition time

**Fig. 6.**
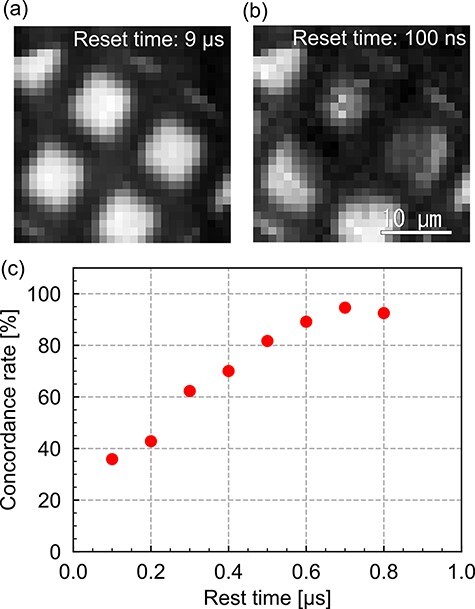
Image disturbance by reset time: (a) normal and (b) distorted TEM images of a copper mesh. The reset times of (a) and (b) are 9 μs and 100 ns, respectively. (c) The concordance rate of the TEM image as a function of the reset time.

The minimum repetition time for STRIEM1 depends on the initialization time to reach the reset voltage for each pixel. In general, the minimum repetition time is limited by the DAQ system because of a long readout time. In the present study, this limitation applies to the sensor chip rather than the DAQ because data are recorded in each pixel. Figures 6(a) and (b) show TEM images, which differ in the reset time, of a 2000 mesh copper TEM grid with a fine square pattern (Ager Scientific), as acquired with STRIEM1. The square pattern is observed in Fig. 6(a) with a reset time of 9 μs. Compared with the pattern in Fig. 6(a), that in Fig. 6(b) with a minimum rest time of 100 ns is blurry.

Figure 6(c) shows the concordance rate for the TEM image as a function of the reset time. The concordance rate was defined as the furcation of the average value of the difference between a reference image and an image having a short reset time. The reference image in Fig. 6(a) with a reset time of 9 μs was used. The concordance rate decreased with decreasing reset time under 1 μs. When we chose the accepted rate of 90%, the minimum reset time was 700 ns. The minimum reset time will be shorter than 700 ns by optimizing the layout pattern of the chip. The CDS reset time, which requires a larger value than the reset time, was set to 800 ns. Therefore, the minimum repetition time of STRIEM1 became 900 ns including the exposure time.

### Demonstration of time-resolved imaging with STRIEM1

**Fig. 7.**
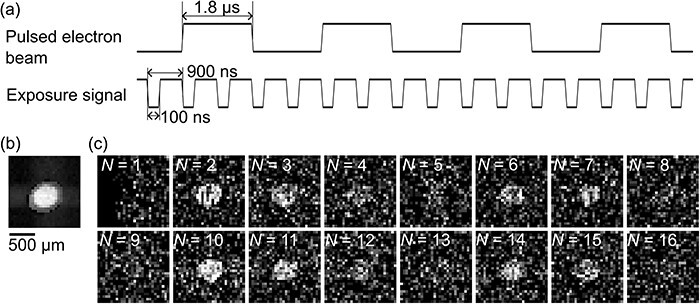
Time-resolved imaging by pulsed electron beams with STRIEM1. (a) A timing diagram of a train of electron pulses and exposure signals. (b) An image of a convergent electron beam. (c) Time-resolved images of convergent electron pulses under the conditions corresponding to subfigure (a). The signals of the pulsed electron beam and the exposure signal are displayed as positive and negative pulses, respectively. The scale bar in (b) represents the size of the detector plane. The number *N* in (c) represents the frame number.

Figure 7 shows demonstration results of time-resolved imaging by convergent electron pulses obtained with STRIEM1. We generated a train of electron pulses with a pulse width of 1.8 μs and recorded the train as a movie consisting of 16 frames at exposure and repetition times of 100 ns and 900 ns, respectively (Fig. 7(a)). The four electron pulses were recorded in 16 frames. The electron beam was converged at the detector plane using projector lenses (Fig. 7(b)). The image of Fig. 7(b), which is a reference image to indicate the beam shape, is displayed as an integrated image consisting of 100 frames with an exposure time of 100 ns. Figure 7(c) shows time-resolved images of the train of convergent electron pulses. The bright-white spot at the center of the images, which represents a convergent electron pulse, was observed in frame numbers 2–3, 6–7, 10–11 and 14–15. This result is in good agreement with the setting of the time diagram in Fig. 7(a). We demonstrated that STRIEM1 can record fast phenomena at the sub-microsecond timescale.

The response time of SOI pixel detectors without circuits and wiring will be limited by the charge collection in the silicon sensor layer with full depletion. The speed of collected electrons in the sensor layer depends on the drift velocity, which is expressed as the product of electron mobility and an external electric field. Assuming that the thickness of the sensor layer with the p-type silicon is 300 μm and that its bias voltage is −250 V, we can estimate the arrival time for collected electrons to the collecting electrode of a pixel as approximately 3 ns. The drift velocity reaches that of the saturated value, which is approximately 1 × 10^7^ cm/s [24], in the above condition. The estimated time is two orders smaller than the minimum repetition time of the current system, which was restricted by the software. Therefore, an SOI pixel detector has the potential to be used for nanosecond imaging if the response of the CMOS circuit can be improved. In addition, we expect a sub-nanosecond response time if the thickness of the sensor layer can be reduced to less than 100 μm.

## Concluding remarks

We developed a prototype SOI pixel detector with 16 analog memories in each pixel, named STRIEM1, along with its dedicated DAQ. The prototype SOI pixel detector, which has 28 × 28 pixels with a pixel size of 50 × 50 μm^2^, was evaluated by TR-TEM in conjunction with the dedicated DAQ. The results showed that the detector can image an object using 30 keV electrons and has a performance equivalent to that of a conventional integration-type direct electron SOI detector. We also demonstrated that the prototype SOI pixel detector can be used in time-resolved imaging at the sub-microsecond scale. The current version of STRIEM has limitations besides the reset time and pixel number. One limitation is that analog output signals are rapidly saturated at the acceleration voltages used in conventional transmission electron microscopes. Another limitation is that the recording length is limited by the number of memories. The former issue has been improved in the next version, in which capacitance is added to control the gain at the sensor node. The latter issue will be improved by a vertical stacking technique [17], which can enlarge the area for memories. In addition, considering the application of the SOI pixel detector to a conventional acceleration voltage in TEM, such as 200 kV, we should reduce the thickness of the sensor layer to compensate for the MTF degradation caused by the scattering of primary electrons in silicon. We expect the STRIEM to enable visualization of high-speed motion via high-speed imaging at the sub-microsecond level.

## Data Availability

The data underlying this article will be shared upon reasonable request to the corresponding author.
